# Opioid Use Disorder in Three Samples of the Lebanese Population: Correlation with Clinical and Genetic Factors

**DOI:** 10.1192/j.eurpsy.2024.244

**Published:** 2024-08-27

**Authors:** K. Chamoun, F. Hajj Moussa Lteif, P. Salameh, H. Sacre, R. Haddad, L. Rabbaa, B. Megarbane, A. Hajj

**Affiliations:** ^1^Laboratoire de Pharmacologie, Pharmacie Clinique et Contrôle de Qualité des Médicaments; ^2^Faculty of Pharmacy, Saint Joseph University, Beirut, Lebanon; ^3^Inserm, UMR-S1144, Université Paris Cité, Paris, France; ^4^INSPECT-LB (Institut National de Santé Publique, d’Épidémiologie Clinique et de Toxicologie-Liban), Beirut; ^5^School of Medicine, Lebanese American University, Byblos, Lebanon; ^6^Department of Primary Care and Population Health, University of Nicosia Medical School, Nicosia, Cyprus; ^7^Faculty of Pharmacy, Lebanese University, Hadat; ^8^Faculty of Medical Sciences, Psychiatry Department, Lebanese University; ^9^Faculty of Medicine, Psychiatry Department, Saint Joseph University, Beirut, Lebanon; ^10^Faculty of Pharmacy, Université Laval; ^11^Oncology Division, CHU de Québec- Université Laval Research Center, Quebec, Canada

## Abstract

**Introduction:**

Opioid Use Disorder (OUD) is a severe and recurrent condition that contributes to a global prevalence of disabilities. Accumulating evidence suggests a potential convergence of clinical and genetic factors underlying OUD.

**Objectives:**

This study explores the clinical and genetic factors associated with OUD in the Lebanese population.

**Methods:**

A cross-sectional study in the Lebanese population included three different groups of participants stratified according to the cut-off of the revised Opioid Risk Tool (ORT-OUD): (1) Low-risk group for OUD (n=513; general population; ORT-OUD score <2.5); (2) High-risk group for OUD (n=87; general population; ORT-OUD score ≥3); (3) a third group consisting of patients clinically diagnosed with OUD according to the DSM-5 (n=46). The survey included sociodemographic information and used validated scales to assess other substance use disorders, sleep disturbances, depression, and anxiety. Genotyping for the *COMT*, *MTHFR*, and *CRY2* genes was conducted for 91 patients using a real-time PCR (Roche^®^). Bivariate and multivariate analyses were conducted to identify the associations between OUD risk and sociodemographic, clinical, and genetic factors.

**Results:**

This study enrolled 646 participants. Multivariate analysis showed significant associations between risk of developing an OUD and cigarette smoking (B=0.583), worse insomnia scores (B=0.074) and Alcohol, Smoking and Substance Involvement Screening Test-alcohol (B=0.053) scores, male gender (B=13.351), lack of education (B=4.159), unemployment (B=7.235), low income (B=11.285), lack of healthcare coverage (B=4.190), neuropsychiatric disorders (B=7.966). Conversely, OUD risk was negatively correlated with the morning chronotype (B=-0.372). Bivariate analysis showed that the *CRY2* AA genotype was significantly associated with a higher risk of OUD; nevertheless, none of the genetic factors remained significant in the multivariable model.

**Conclusions:**

This study identified several sociodemographic, clinical, and genetic factors that could potentially increase the risk of developing OUD in the Lebanese population. Further research is needed to clarify risk factors and underlying mechanisms, enabling the development of more effective prevention strategies.

**Disclosure of Interest:**

None Declared

